# Author Correction: Epidemiological drivers of transmissibility and severity of SARS-CoV-2 in England

**DOI:** 10.1038/s41467-023-44062-9

**Published:** 2023-12-07

**Authors:** Pablo N. Perez-Guzman, Edward Knock, Natsuko Imai, Thomas Rawson, Cosmo Nazzareno Santoni, Joana Alcada, Lilith K. Whittles, Divya Thekke Kanapram, Raphael Sonabend, Katy A. M. Gaythorpe, Wes Hinsley, Richard G. FitzJohn, Erik Volz, Robert Verity, Neil M. Ferguson, Anne Cori, Marc Baguelin

**Affiliations:** 1 MRC Centre for Global Infectious Disease Analysis and Abdul Latif Jameel Institute for Disease and Emergency Analytics (J-IDEA), School of Public Health, Imperial College London, London, UK; 2 Adult Intensive Care Unit, Royal Brompton Hospital, London, UK; 3 Department of Surgery and Cancer, Faculty of Medicine, Imperial College London, London, UK; 4 Department of Engineering, Division of Electrical Engineering, University of Cambridge, Cambridge, UK; 5 National Institute for Health Research (NIHR) Health Protection Research Unit (HPRU) in Modelling and Health Economics, London, UK; 6 Centre for Mathematical Modelling of Infectious Diseases, Department of Infectious Disease Epidemiology, London School of Hygiene & Tropical Medicine, London, UK

**Keywords:** Infectious diseases, Computational models, Epidemiology, SARS-CoV-2

Correction to: *Nature Communications* 10.1038/s41467-023-39661-5, published online 17 July 2023

The original version of this Article contained several errors in the Abstract, Results, Figures, and Supplementary Information. This occurred because incorrect parameter values were used in the code.

The code linked in the Code availability statement in the Article is correct: https://github.com/mrc-ide/sarscov2-severity-england. The previous incorrect version of the code is available at: https://github.com/mrc-ide/sarscov2-severity-england/releases/tag/v1.0.0. The current and previous versions of the code can be compared at: https://github.com/mrc-ide/sarscov2-severity-england/pull/2.

The original version of this Article contained errors in the Abstract and Results:

1. Page 1, Abstract: “basic reproduction number 8.3 (95% credible interval (CrI) 7.7–8.8)” has been corrected to “basic reproduction number 8.4 (95% credible interval (CrI) 7.8–9.1)”
2. Page 1, Abstract: “basic infection fatality ratio (2.9%, 95% CrI 2.7–3.2)” has been corrected to “basic infection fatality ratio (3.0%, 95% CrI 2.8–3.2)”
3. Page 1, Abstract: “Delta (2.2%, 95% CrI 2.0–2.4)” has been corrected to “Delta (2.1%, 95% CrI 2.0–2.4)”
4. Page 3, Results, paragraph starting “Viral evolution tends to select for more transmissible lineages”: “credible interval (CrI) 2.4–2.7 for the initial Wildtype virus, to 4.2 (95% CrI 3.9–4.4), 7.0 (95% CrI 6.5–7.4) and 8.3 (95% CrI 7.7–8.8)” has been changed to “credible interval (CrI) 2.4–2.8 for the initial Wildtype virus, to 4.2 (95% CrI 4.0–4.6), 7.2 (95% CrI 6.7–7.8) and 8.4 (95% CrI 7.8–9.1)”
5. Page 3, Results, paragraph starting “NPIs were first introduced in England in mid-March 2020”: “7.8% (95% CrI 7.3–8.3)” has been changed to “7.9% (95% CrI 7.4–8.3)”.
6. Page 3, Results, paragraph starting “The basic IHR of SARS-CoV-2 increased from 2.2%”: “3.3% (95% CrI 3.0–3.5) for Alpha, 4.2% (95% CrI 3.8–4.4) for Delta, but subsequently decreased to 3.4% (95% CrI 3.1–3.8)” has been changed to “3.4% (95% CrI 3.1–3.6) for Alpha, 4.2% (95% CrI 4.1–4.4) for Delta, but subsequently decreased to 3.1% (95% CrI 2.7–3.3)”
7. Page 3, Results, paragraph starting “The basic IHR of SARS-CoV-2 increased from 2.2%”:”basic HFR of 48.5% (95% CrI 44.3–52.9), lower for Wildtype and Delta, at 32.6% (95% CrI 30.7–34.4) and 32.6% (95% CrI 29.2–36.6), respectively, and lowest for Omicron (BA.1), at 14.3% (95% CrI 11.3–17.1)” has been changed to “basic HFR of 50.3% (95% CrI 46.7–53.6), lower for Wildtype and Delta, at 33.0% (95% CrI 30.7–35.3) and 32.3% (95% CrI 27.4–35.8), respectively, and lowest for Omicron (BA.1), at 15.5% (95% CrI 13.1–18.8)”
8. Page 3, Results, paragraph starting “The basic IHR of SARS-CoV-2 increased from 2.2%”: “Alpha variant at 2.9% (95% CrI 2.7–3.2), followed by Delta at 2.2% (95% CrI 2.0–2.4)” has been changed to “Alpha variant at 3.0% (95% CrI 2.8–3.2), followed by Delta at 2.1% (95% CrI 1.9–2.4)”
  The errors in the Abstract and Results have been corrected in both the PDF and HTML versions of the Article.
  The original version of this Article contained errors in Figures:
9. The original version of this Article contained errors in Figure 1. The correct version of Figure 1 is:
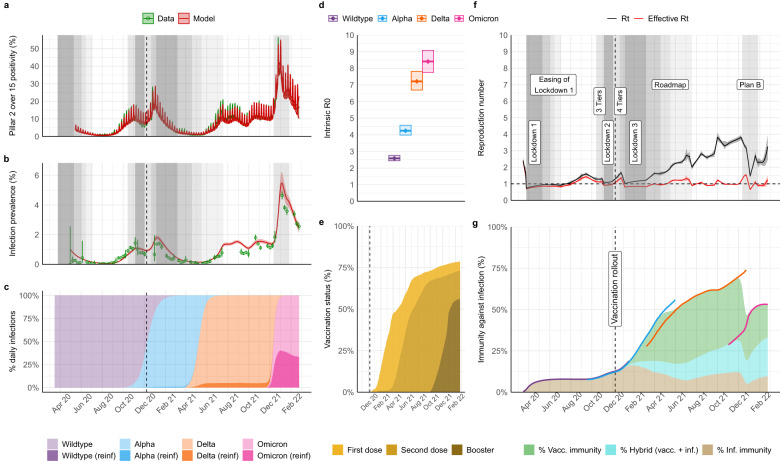
  which replaces the previous incorrect version:
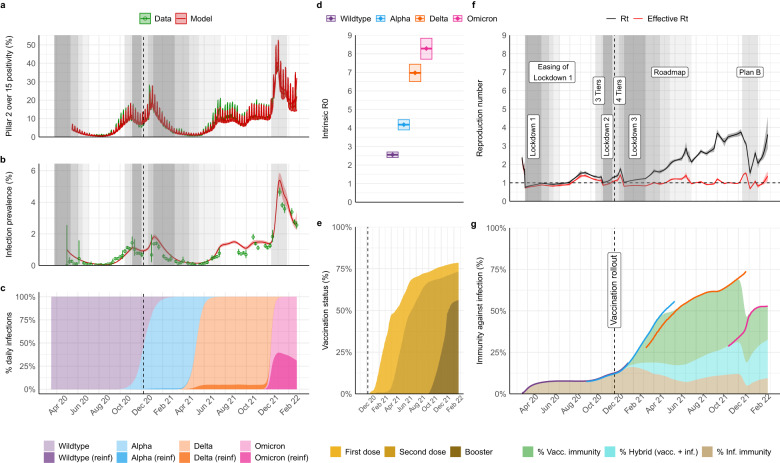
10. The original version of this Article contained errors in Figure 2. The correct version of Figure 2 is:
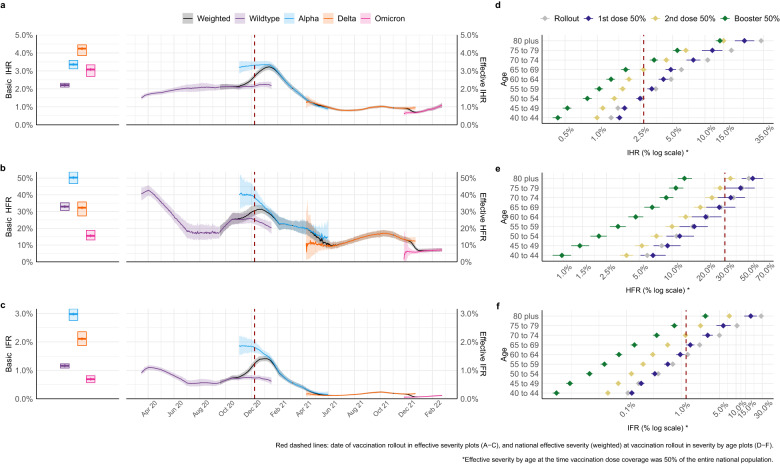
  which replaces the previous incorrect version:
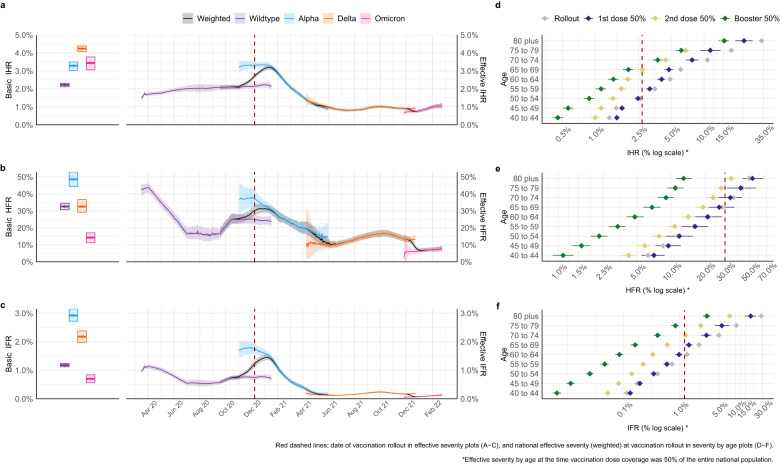
  The errors in Figures 1 and 2 have been corrected in both the PDF and HTML versions of the Article.
11. The original version of the Supplementary Information associated with this Article contained errors in Tables S2, S3, S18-21, S23, S24; and Figures S2, S8-10, S12-50. It also contained typographical errors in equation 18, 22, 40, 270, and 271; and in sections 4.7 and 4.11.

The HMTL has been updated to include a corrected version of the Supplementary Information; the original incorrect version of the Supplementary Information can be found as Supplementary Information associated with this Correction.

## Supplementary information

Updated Supplementary Information

Incorrect Supplementary Information

