# Supplementary material for: Epidemiological drivers of transmissibility and severity of SARS-CoV-2 in England
Source: Nat Commun. 2023 Jul 17;14:4279. doi: 10.1038/s41467-023-39661-5 (PMC10352350; doi:10.1038/s41467-023-39661-5)
Supplement: Supplementary file 3 — Reporting Summary [file 41467_2023_39661_MOESM3_ESM.pdf]

nature portfolio

Corresponding author(s): Marc BAGUELIN

Last updated by author(s): Jun 19, 2023

Reporting Summary

Nature Portfolio wishes to improve the reproducibility of the work that we publish. This form provides structure for consistency and transparency in reporting. For further information on Nature Portfolio policies, see our Editorial Policies and the Editorial Policy Checklist.

Statistics

For all statistical analyses, confirm that the following items are present in the figure legend, table legend, main text, or Methods section.

n/a	Confirmed	
☐	☒	The exact sample size (*n*) for each experimental group/condition, given as a discrete number and unit of measurement
☒	☐	A statement on whether measurements were taken from distinct samples or whether the same sample was measured repeatedly
☐	☒	The statistical test(s) used AND whether they are one- or two-sided<br>*Only common tests should be described solely by name; describe more complex techniques in the Methods section.*
☐	☒	A description of all covariates tested
☐	☒	A description of any assumptions or corrections, such as tests of normality and adjustment for multiple comparisons
☐	☒	A full description of the statistical parameters including central tendency (e.g. means) or other basic estimates (e.g. regression coefficient) AND variation (e.g. standard deviation) or associated estimates of uncertainty (e.g. confidence intervals)
☐	☒	For null hypothesis testing, the test statistic (e.g. *F*, *t*, *r*) with confidence intervals, effect sizes, degrees of freedom and *P* value noted<br>*Give P values as exact values whenever suitable.*
☐	☒	For Bayesian analysis, information on the choice of priors and Markov chain Monte Carlo settings
☒	☐	For hierarchical and complex designs, identification of the appropriate level for tests and full reporting of outcomes
☒	☐	Estimates of effect sizes (e.g. Cohen's *d*, Pearson's *r*), indicating how they were calculated

Our web collection on statistics for biologists contains articles on many of the points above.

Software and code

Policy information about availability of computer code

Data collection	The data, code and scripts used to create the results in this paper are available in (https://github.com/mrc-ide/sarscov2-severity-england). The study used secondary data and all the data pipelines were built in the R environment.
Data analysis	The data, code and scripts used to create the results in this paper are available in (https://github.com/mrc-ide/sarscov2-severity-england). The data, scripts and information on versions are provided there. Regarding the R packages developed by the team to built the models the following versions have been used : dust v0.13.2, mcstate v0.9.14,sircovid v0.14.11, and spimalot v0.8.24.

For manuscripts utilizing custom algorithms or software that are central to the research but not yet described in published literature, software must be made available to editors and reviewers. We strongly encourage code deposition in a community repository (e.g. GitHub). See the Nature Portfolio guidelines for submitting code & software for further information.

Data

Policy information about availability of data

All manuscripts must include a data availability statement. This statement should provide the following information, where applicable:

- Accession codes, unique identifiers, or web links for publicly available datasets
- A description of any restrictions on data availability
- For clinical datasets or third party data, please ensure that the statement adheres to our policy

Ethics permission was sought for the study through the standard ethical review processes of Imperial College London (London, UK), and was approved by the

university's research governance and integrity team (Imperial College Research Ethics Committee reference 21IC6945). Patient consent was not required as the research team accessed fully anonymised data only, which were collected as part of routine public health surveillance activities by the UK Government. The data, code and scripts used to create the results in this paper are available in (https://github.com/mrc-ide/sarscov2-severity-england)

Research involving human participants, their data, or biological material

Policy information about studies with human participants or human data. See also policy information about sex, gender (identity/presentation), and sexual orientation and race, ethnicity and racism.

Reporting on sex and gender	The model used for the analysis is a compartmental model stratified by age, regions, vaccination status, and infection history. Compartments of the models thus include individuals from all sexes and genders. The data streams used to fit also include all sexes and genders. We do provide all data necessary to reproduce the results but not provide data at the individual level or at a level of disaggregation lower than needed for the model due to disclosure closure. All results thus apply to all sexes and genders.
Reporting on race, ethnicity, or other socially relevant groupings	N/A
Population characteristics	Population was stratified by 5-years age groups in our study with a separate group for all individuals above 80 years old. Any over break down was driven by the data provided to us and are explained in the Supplementary Material when relevant.
Recruitment	This study was built from secondary data and thus we did not have to recruit human participants. Part of the data comes from routine surveillance and the others come from specific studies, whose references, relevant publications can be found in the main manuscript or the SI.
Ethics oversight	Ethics permission was sought for the study through the standard ethical review processes of Imperial College London (London, UK), and was approved by the university's research governance and integrity team (Imperial College Research Ethics Committee reference 21IC6945). Patient consent was not required as the research team accessed fully anonymised data only, which were collected as part of routine public health surveillance activities by the UK Government.

Note that full information on the approval of the study protocol must also be provided in the manuscript.

Field-specific reporting

Please select the one below that is the best fit for your research. If you are not sure, read the appropriate sections before making your selection.

☒ Life sciences ☐ Behavioural & social sciences ☐ Ecological, evolutionary & environmental sciences

For a reference copy of the document with all sections, see nature.com/documents/nr-reporting-summary-flat.pdf

Life sciences study design

All studies must disclose on these points even when the disclosure is negative.

Sample size	The data includes all data available for England.
Data exclusions	No data was excluded from the analysis.
Replication	All simulations and inference can be reproduced using the code provided there: https://github.com/mrc-ide/sarscov2-severity-england
Randomization	We did not collect primary data. The methodologies used for the collection of the different data we used will thus depend on the specific protocol of these studies. Also a large part of the data streams is from routine surveillance thus including all reports available.
Blinding	We did not collect primary data. As for the randomization the methodologies used for the collection of the different data we used will thus depend on the specific protocol of these studies.

Reporting for specific materials, systems and methods

We require information from authors about some types of materials, experimental systems and methods used in many studies. Here, indicate whether each material, system or method listed is relevant to your study. If you are not sure if a list item applies to your research, read the appropriate section before selecting a response.

Materials & experimental systems

n/a	Involved in the study
☒	☐ Antibodies
☒	☐ Eukaryotic cell lines
☒	☐ Palaeontology and archaeology
☒	☐ Animals and other organisms
☒	☐ Clinical data
☒	☐ Dual use research of concern
☒	☐ Plants

Methods

n/a	Involved in the study
☒	☐ ChIP-seq
☒	☐ Flow cytometry
☒	☐ MRI-based neuroimaging
